# The next generation of dental treatments: Leveraging smart-responsive nanomaterials for unique oral environments

**DOI:** 10.1016/j.mtbio.2025.102057

**Published:** 2025-07-05

**Authors:** Chenying Cui, Jingyu Yan, Lihong Zhou, Yurong Xu, Guning Wang, Xiuping Wu, Bing Li

**Affiliations:** a Shanxi Medical University School and Hospital of Stomatology, Taiyuan, 030001, Shanxi, China; b Shanxi Province Key Laboratory of Oral Diseases Prevention and New Materials, Taiyuan, 030001, Shanxi, China; c Academy of Medical Sciences, Shanxi Medical University, Taiyuan, 030001, Shanxi, China

**Keywords:** Oral microenvironment, Smart-responsive nanomaterials, Tooth tissue, Periodontal tissue, Tissue regeneration

## Abstract

Oral diseases are highly prevalent worldwide and are a burden to public health systems and economic resources. They often destroy oral tissues, impairing essential functions such as mastication and speech. Conventional therapeutic strategies face several challenges, including poor biocompatibility and limited regenerative outcomes, and are frequently constrained by the complex and dynamic oral microenvironment. Smart-responsive nanomaterials provide effective solutions by dynamically reacting to key pathological features of the oral milieu, such as dysbiosis, oxidative stress, acidic pH, and immune imbalance. These materials facilitate targeted tissue repair and regeneration through the precise, stimuli-responsive release of therapeutic agents. In this review, we summarized the unique physiological and pathological characteristics of the oral microenvironment, discussed the fundamental response mechanisms of nanomaterials sensitive to physical, chemical, and biological stimuli, and analyzed the composition and design of emerging nanoplatforms. We highlighted their applications in tooth and periodontal tissue regeneration and discussed current challenges and key considerations in clinical translation to provide a theoretical foundation for future clinical advancements in oral regenerative medicine.

## Highlights

• Smart-responsive nanomaterials adapt to the dynamic oral pathological microenvironment.
• These materials enable targeted and stimulus-controlled release of therapeutic agents.
• Physical, chemical, and biological stimuli are utilized for precise regenerative responses.
• Applications in tooth and periodontal tissue regeneration are comprehensively reviewed.
• Challenges in clinical translation and future research directions are critically discussed.

## Graphical abstract

**Figure:**
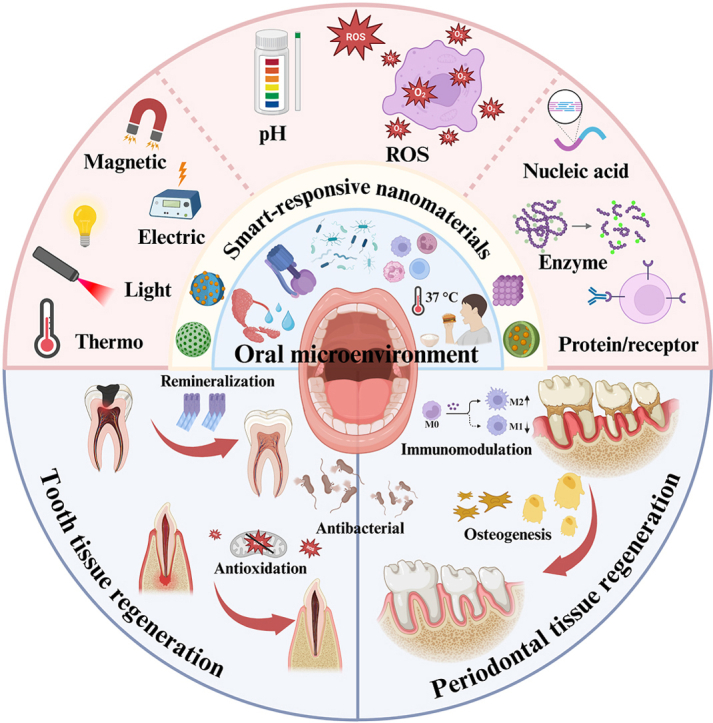
Smart nanomaterials responsive to the oral microenvironment facilitate oral tissue regeneration.

## 1 Introduction

Oral diseases are highly prevalent worldwide [1] and pose significant medical and economic burdens while impairing the quality of life and overall health of individuals. Epidemiological data indicate that about 3.69 billion people globally were affected by major oral diseases in 2021. Among them, untreated dental caries in permanent teeth and severe periodontitis are the most common, with global age-standardized prevalence rates of about 27,500 and 12,500 cases per 100,000 people, respectively [2]. Oral diseases include a wide range of acute and chronic conditions [3] that involve structural and functional damage to teeth and oral tissues. These impairments can hinder key oral functions such as mastication and speech and may even trigger systemic inflammatory responses [4]. Conventional treatment approaches, such as biomaterial implantation, guided tissue regeneration, and autologous or allogeneic tissue transplantation, provide partial relief in managing tissue defects. However, these methods are often limited by issues such as poor biocompatibility, high risk of immune rejection, and insufficient efficiency of regeneration. These limitations highlight that more effective therapeutic strategies need to be developed.

Owing to their high surface area, superior mechanical properties, tunable degradation profiles, and excellent bioactivity, nanomaterials have shown great promise in tissue engineering [5,6]. In the field of dentistry, nanotechnology has triggered a transformative shift, reshaping our understanding of oral health and revolutionizing the diagnosis and treatment of related diseases. However, the efficacy of these nanomaterials is significantly reduced by the complex pathological microenvironment surrounding oral lesions. Factors such as microbial dysbiosis, acidic pH, oxidative stress, and immune dysregulation greatly hinder their performance. The highly mobile, moist, and open nature of the oral cavity further undermines the stability and functionality of conventional nanomaterials, ultimately affecting the diagnosis, treatment, and prognosis of oral diseases [7,8].

In contrast, smart-responsive nanomaterials can sense and adapt to local physiological or pathological changes, thereby creating a more favorable environment for the repair and regeneration of tissues. Based on their response mechanisms, stimuli-responsive nanomaterials can be classified into physical (e.g., temperature and electromagnetic fields), chemical (e.g., pH and redox), and biological (e.g., enzymes and cellular signals) categories. These materials perform adaptive functional modulation by sensing endogenous biological cues or external stimuli, thereby allowing targeted delivery and controlled release of bioactive agents to dynamically regulate tissue regeneration. For example, pH-sensitive systems release therapeutic agents in acidic microenvironments [9]; photo-, electro-, and magneto-responsive materials can activate cellular functions under external stimulation [10]; and multi-modal platforms integrate various stimuli to achieve synergistic regenerative outcomes.

Conventional treatment approaches for oral diseases rely primarily on systemic drug administration or local surgical intervention, which are often associated with poor targeting specificity, increased risk of drug resistance, and significant side effects. Similarly, traditional regenerative strategies rely on exogenous cell transplantation or single bioactive factors, which are restricted by low cell viability, poor tissue integration, and insufficient regenerative capacity. In contrast, smart-responsive nanomaterials offer multiple advantages, including precise targeting, dynamic responsiveness, and controlled release [11]. These materials respond to the specific microenvironment of the oral cavity, allowing site-, time-, and dose-specific drug delivery and biological regulation, thereby significantly improving therapeutic outcomes and efficiency of tissue regeneration [12]. Some smart nanomaterials also integrate diagnostic and therapeutic functionalities, allowing real-time monitoring and precise intervention at disease sites [13,14]. Early-stage detection of dental conditions via smart nanodiagnostic systems facilitates tailored therapeutic interventions, thereby decreasing the reliance on invasive dental procedures [3]. By simulating the gradient of physiological signals and regulating behaviors such as cell chemotaxis, adhesion, proliferation, and differentiation, a regenerative microenvironment conducive to tissue reconstruction can be created, which can effectively overcome the bottleneck of insufficient regeneration in traditional treatments [15]. These materials often possess self-regulating and self-limiting release mechanisms, which enhance therapeutic efficacy while reducing the frequency of clinical interventions. This not only alleviates the burden on patients but also improves their compliance and satisfaction [3,16]. Overall, smart-responsive nanomaterials demonstrate potential for multidimensional innovation in precision therapy and tissue engineering, offering promising strategies to overcome the limitations of conventional treatments and facilitating efficient, targeted interventions and functional regeneration for complex oral diseases [17].

**Scheme 1:**
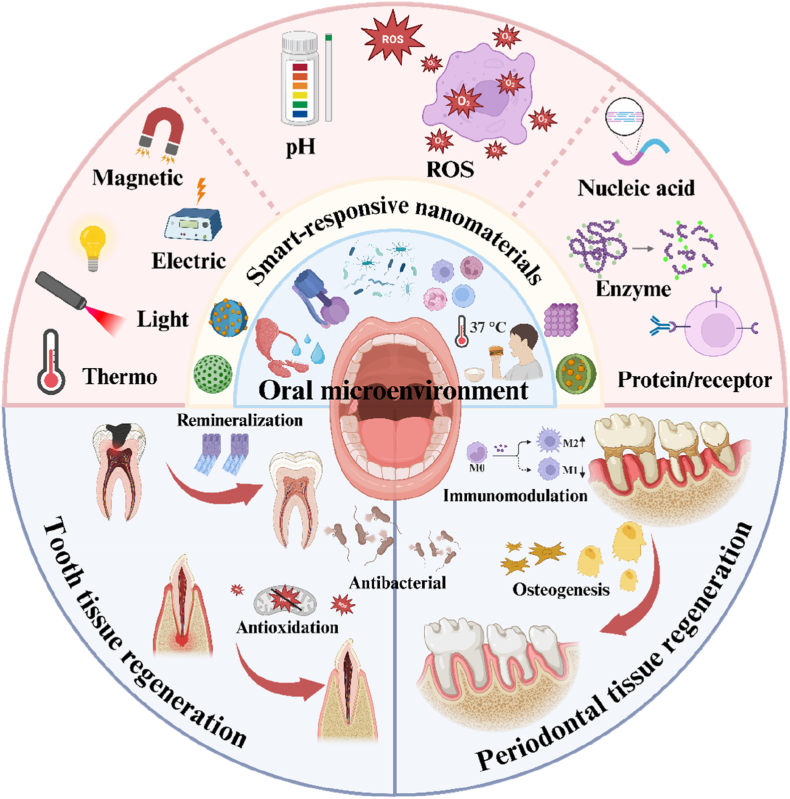
Smart nanomaterials responsive to the oral microenvironment facilitate oral tissue regeneration. Created in BioRender. (2025) https://BioRender.com.

Many reviews in recent years have focused on functional materials used for the treatment of oral diseases. For example, Zhang et al. emphasized the design and clinical potential of collagen-based biomaterials in the reconstruction of oral and craniofacial tissues [18]. Another review by Zhang et al. discussed the design, synthesis, and dental applications of engineered gold nanostructures, highlighting their therapeutic roles in caries, periodontitis, peri-implantitis, and oral mucosal diseases [3]. Jia et al. investigated structural design strategies for polymer-based biomaterials for oral disease management [8], whereas Li et al. systematically summarized recent advancements in functional hydrogels for treating hard dental tissue and pulp-related conditions [19]. Other reviews have addressed the progress of stimuli-responsive materials and their potential in precision medicine [[20], [21], [22], [23], [24], [25]]. However, most of these studies focused on specific material types or isolated applications [[26], [27], [28]], with limited attention given to smart biomaterials that can respond adaptively to the complex and dynamic microenvironment of the oral cavity. To address this issue, in this review, we systematically summarized recent progress in the development of smart stimuli-responsive nanomaterials for modulating the unique pathological microenvironment of the oral cavity to promote tissue regeneration (Scheme 1). We first outlined the inherent characteristics of oral tissues, focusing on the pathological microenvironment associated with tissue damage. Next, we categorized and discussed the response mechanisms of microenvironment-sensitive nanomaterials, including physical, chemical, and biological stimuli. We then illustrated their application strategies and elucidated their underlying mechanisms in representative cases of tooth and periodontal tissue regeneration. Finally, we highlighted future directions and clinical translation challenges associated with these advanced materials. By identifying research hotspots and technological bottlenecks, we aimed to provide a theoretical foundation and practical guidance for further exploration and application in the field of oral regenerative medicine.

## 2 The intrinsic microenvironment of the oral cavity and its changes under pathological injury conditions

The digestive and respiratory systems start in the oral cavity, the microenvironment of which plays a key role in maintaining physiological homeostasis and tissue regeneration. Under normal conditions, the oral microenvironment is regulated by factors such as temperature, saliva, the microbiota, the immune system, and mechanical stress, thus maintaining a dynamic equilibrium [29]. However, under pathological conditions or after an injury, this microenvironment undergoes significant changes, affecting the functions of cells, inflammatory responses, and the repair process. Therefore, a comprehensive understanding of the microenvironmental characteristics of oral tissues and their changes under pathological conditions is necessary for the application of smart-responsive nanomaterials in tissue regeneration.

**Fig. 1:**
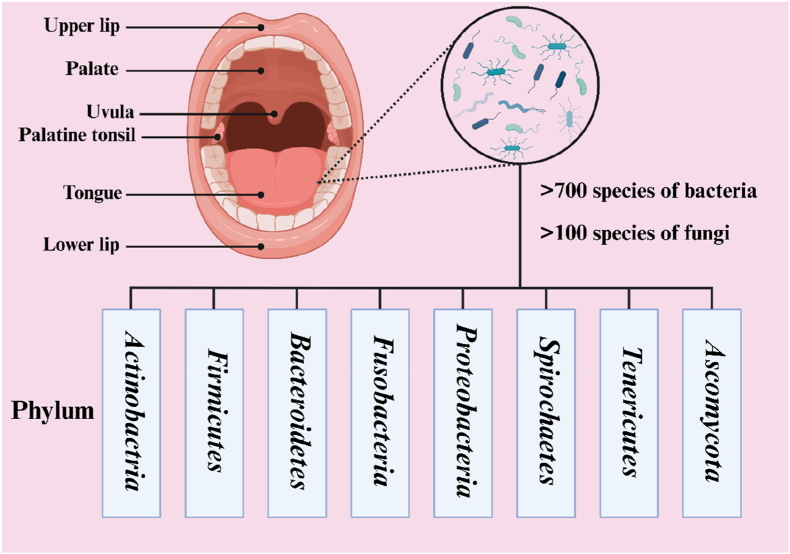
Schematic representation of the oral cavity microbiota distribution and microbial diversity. Created in BioRender. (2025) https://BioRender.com.

Under physiological conditions, the temperature of the oral cavity is usually maintained at 36.5–37.5 °C, which is close to the human core body temperature. This relatively stable temperature provides a suitable environment for the normal metabolism of cells and the activity of various enzymes in the oral cavity. Saliva not only provides moisture and nutrients but also maintains the pH of the oral cavity between 6.5 and 7.0 through its buffering system (HCO_3_^−^ and PO_4_^3−^), thereby stabilizing cellular metabolism. Another important characteristic of the oral microenvironment is the coexistence of more than 700 bacterial species (Fig. 1) [30], constituting one of the most complex and dense microbial ecosystems in the human body, including bacteria, archaea, fungi, protozoa, and viruses [31]. Oral tissues are situated in a highly dynamic environment and are constantly exposed to essential mechanical activities such as mastication, swallowing, and speech. Mechanical loading can stimulate bone regeneration and maintain the dynamic balance of bone mass. The immune system plays an important role in the homeostasis of oral tissues. It is composed of mucosal barriers, immune cells, and immune molecules and can rapidly sense the invasion of pathogens and regulate microbial balance through low-grade inflammation, preventing excessive immune reactions from damaging host tissues.

Under pathological conditions, disruption of oral homeostasis can hinder tissue repair capabilities and even exacerbate disease progression. Dysbiosis can promote the production of virulence factors and metabolic byproducts by pathogenic microorganisms, triggering a cascade of diseases. For example, the initial pathogenic factors of caries and periodontitis involve the formation of bacterial biofilm on the tooth surface, which is embedded within an extracellular polysaccharide (EPS)-rich matrix that shields the internal microbiota from attacks by drugs and the immune system. *Streptococcus* species, such as *S. oralis*, act as pioneer colonizers, followed by other oral plaque bacteria, such as *S. mutans*, which metabolize sugars to produce acids and EPS, thereby suppressing salivary buffering and enhancing resistance to antimicrobial agents [32,33]. The resulting acidic byproducts decrease the local pH to below 5.0, leading to demineralization of enamel, degradation of cementum, and periodontal tissue damage. Infections caused by *S. aureus* in implants can lead to mucositis and bone resorption, with severe cases requiring the removal of implants and subsequent surgery [34]. Additionally, microbial dysbiosis-induced systemic inflammation can increase the risk of other complex diseases, including diabetes, cancer, and myocardial infarction [35,36].

Reactive oxygen species (ROS) are byproducts of cellular metabolism, particularly the mitochondrial respiratory chain, and include hydrogen peroxide (H_2_O_2_), superoxide anions (O_2_**^•^**^–^), monoclinic oxygen (^1^O_2_), and hydroxyl radicals (**•**OH). Excessive accumulation of ROS can induce oxidative stress, triggering inflammation, apoptosis, and tissue damage. ROS also significantly influences the progression of diseases such as pulpitis and periodontitis by regulating the expression of inflammation-related genes and modulating the polarization of macrophages [37]. Immune imbalance is another important mechanism: M1 macrophages release proinflammatory factors, including inducible nitric oxide synthase (iNOS) and tumor necrosis factor-alpha (TNF-*α*), which promote osteoclast activation, whereas a reduction in M2 macrophages weakens repair responses, leading to prolonged inflammation [38]. Epidemiological studies also showed that periodontitis may have detrimental effects on various systemic diseases, including diabetes, Alzheimer's disease, COVID-19, and certain malignancies [39].

Hypoxia is a major challenge in oral tissue regeneration following injury. Under traumatic or ischemic conditions, such as oral ulcers or periodontal defects, endothelial dysfunction promotes the adhesion of immune cells, lipid accumulation, an increase in vascular permeability, and a decrease in elasticity [40], collectively leading to local hypoxia. This hypoxic microenvironment impairs the functions of osteoblasts and fibroblasts. Additionally, an increase in the activity of matrix metalloproteinases accelerates the degradation of the extracellular matrix (ECM), disrupting cell adhesion and signaling pathways and thereby compromising tissue regeneration. In chronic inflammatory conditions such as periodontitis, the degradation of the ECM exceeds its synthesis, making regeneration of the periodontal ligament (PDL), alveolar bone, and soft tissues difficult [41].

To summarize, the microenvironment of oral tissues differs significantly between healthy and pathological states, strongly influencing tissue regeneration and repair. Key pathological features, such as dysbiosis, chronic inflammation, oxidative stress, hypoxia, and ECM degradation, greatly reduce the effectiveness of conventional treatment strategies. The highly dynamic and moist environment of the oral cavity hinders the stable adhesion of drug delivery systems and tissue-repair materials, limiting the retention time and compromising biological functionality [8]. To address these problems, smart-responsive nanomaterials offer the ability to precisely sense and modulate dynamic changes in the oral microenvironment, thereby providing unique advantages in regenerative applications [42]. In the following sections, we discussed the underlying mechanisms of various types of smart nanomaterials and their recent advancements in promoting oral tissue repair.

## 3 Smart response strategy

The major advantages of smart nanomaterials include their highly tunable surface chemistry and structural versatility. By grafting functional polymers, incorporating stimulus-responsive ligands, or engineering hierarchical architectures, these materials can be precisely programmed to recognize and respond to specific environmental signals [17,22,43]. For example, thermo-responsive polymers can undergo phase transitions triggered by temperature changes [17], pH-sensitive moieties facilitate degradation in acidic microenvironments [44], and biomolecular modifications, such as cell-targeting peptides [45], facilitate selective cellular interactions. This modification not only allows the fine-tuning of surface functionalities and internal structures but also enables the dynamic control of biological activities, including the spatiotemporal release of therapeutic agents and the regulation of cell behavior. Through deliberate functionalization strategies that impart sensitivity to distinct physiological or pathological stimuli, smart nanomaterials can be engineered to exhibit various responsive behaviors. These mechanisms can be systematically classified into four main categories: physical, chemical, biological, and multi-modal responses.

In this section, we discussed the fundamental principles underlying the responsive mechanisms of smart nanomaterials, focusing on how physical stimuli (e.g., temperature, light, and ultrasound), chemical triggers (e.g., pH and redox conditions), biological cues (e.g., enzyme activity and receptor recognition), and their synergistic multi-modal interactions regulate the behavior of nanomaterials in the oral microenvironment. By systematically evaluating these mechanisms, we provided a detailed understanding of how smart nanomaterials can be rationally engineered to meet the complex demands related to the treatment of oral diseases and tissue regeneration.

### 3.1 Physical stimulus response

Physical stimulus-responsive mechanisms use external energy inputs, such as heat, light, electricity, magnetism, or ultrasound, to remotely induce controlled structural or phase transitions in smart nanomaterials. These materials are particularly advantageous for enabling precise external manipulation and energy-based therapies. Thermo-responsive materials utilize intraoral temperature to induce gelation for sustained release or drug activation, making them suitable for applications such as pulpitis treatment [46]. Light-responsive materials can be activated by specific wavelengths of light to achieve highly spatiotemporally precise antimicrobial photodynamic therapy (aPDT), applicable to conditions including periodontitis, peri-implantitis, and dental caries [47]. Light-curable resins also represent a classic example of photo-responsive applications in dental practice [48].

#### 3.1.1 Temperature response

Thermo-responsive materials refer to certain polymers that can undergo a reversible physical state transition under a temperature change, which often manifests as a change from the solution state to the gel state. Among them, thermosensitive hydrogels constructed based on the block copolymers have become a research hotspot, with typical representatives such as Pluronic F127 (PEO-PPO-PEO) [49] and poly(D,L-lactide)-poly(ethylene glycol)-poly(D,L-lactide) (PLA-PEG-PLA, PPP) [50] triblock copolymers. The thermosensitive mechanism is mainly based on the shift in the hydrophilic/hydrophobic equilibrium. At low temperatures, the hydrophilic segments dominate and form a homogeneous solution; at body temperatures, the hydrophobic segments are dehydrated and they self-assemble into micelles, which further aggregate to form a three-dimensional network and achieve gelation. This process involves the regulation of the critical micelle concentration and critical gel temperature. These materials have adequate injectability, biocompatibility, and *in situ* gel-forming ability and can achieve intelligent release of biological factors, which are extensively used in oral tissue regeneration. Especially in endodontic restorations [50], thermo-responsive hydrogels are easy to inject at room temperature and gel quickly at body temperature. They adapt to the complex cavity structure and combine the advantages of easy operation and precise treatment.

#### 3.1.2 Light response

**Fig. 2:**
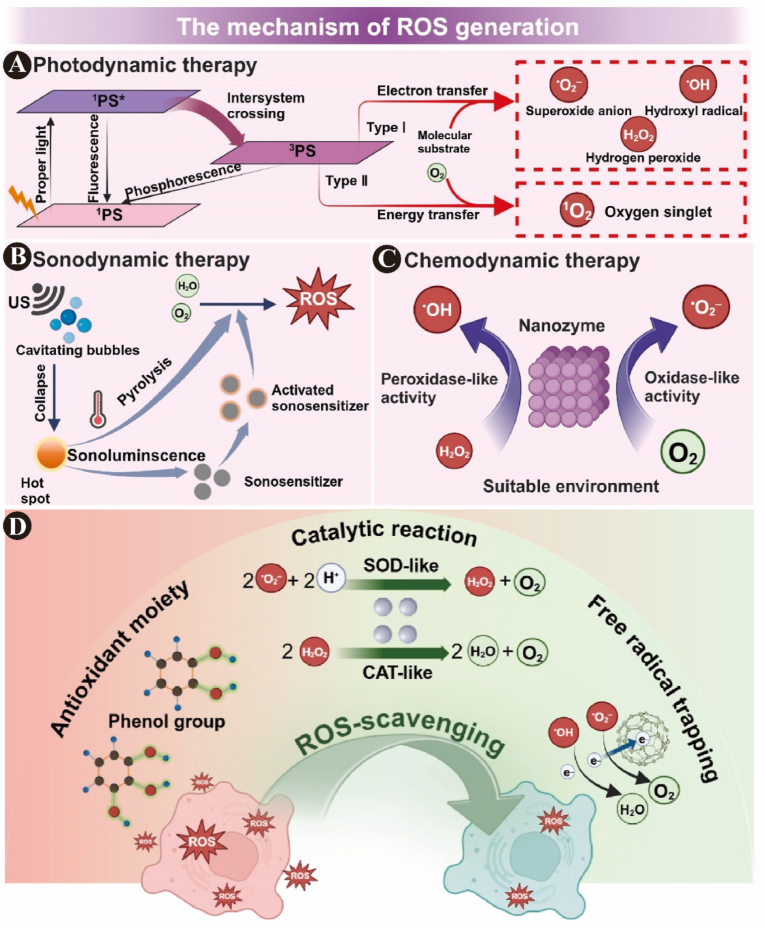
Mechanisms of ROS generation and scavenging. Three major therapeutic strategies for ROS generation: A) PDT produces ROS via light-activated PSs through Type I (electron transfer) or Type II (energy transfer) mechanisms. B) SDT relies on ultrasound-induced cavitation and sonoluminescence to activate sonosensitizers and generate ROS. C) CDT utilizes nanozymes with POD- or oxidase-like activity to catalyze ROS production under specific conditions. D) Strategies for ROS scavenging by nanomaterials, including antioxidant moieties (e.g., phenol groups), enzyme-mimetic catalytic reactions (SOD-like and CAT-like), and free radical trapping, help maintain redox balance and protect against oxidative stress [16]. Reproduced with permission from Ref. [16] Copyright 2024, Elsevier.

Phototherapy, a non-invasive, broad-spectrum antibacterial, fast-acting, and nonresistance-inducing treatment with good temporal and spatial controllability, mainly includes PDT and photothermal therapy (PTT), which are widely used in the field of medicine. PTT uses photothermal agents to generate heat under near-infrared (NIR) irradiation, which increases the local temperature to over 50 °C, thereby killing bacteria and eradicating biofilms. However, the antimicrobial effects of PTT are affected by temperature fluctuations, and bacteria may recur once the temperature is lowered, while high temperatures may also damage normal tissues [51]. PDT activates photosensitizers (PSs) through specific wavelength lasers, inducing the production of ROS, which triggers disorders in the structure and function of cells [52,53]. This process begins with the excitation of the PS from its ground state to an excited singlet state, followed by an intersystem transition to a more stable triplet state. In this triplet state, PSs can return to their ground state via two primary mechanisms (Fig. 2A) [54]. In Type I PDT, triplet-state PS undergoes electron transfer with the surrounding substrates, generating a series of ROS, including O_2_^•–^, H_2_O_2_, and •OH. In contrast, Type II PDT involves direct energy transfer from the excited PS to O_2_, producing ^1^O_2_. These two pathways can occur simultaneously, although Type I reactions are generally favored under hypoxic conditions [54,55]. As long as the PS remains active, it can keep absorbing light energy and generate ROS, thereby enhancing therapeutic efficacy.

Compared to monotherapy, nanotechnology-based dual phototherapy strategies are characterized by multifunctional integration and often show stronger therapeutic synergies, higher antimicrobial efficiency, and lower drug dose and laser power [56]. For example, indocyanine green (ICG), the U.S. Food and Drug Administration (FDA)-approved PS, can produce ROS and thermal effects under NIR excitation, effectively enhancing local antimicrobial capacity [34]. The tumor-targeting capability of phototherapy also endows it with broad application prospects in cancer treatment, enabling localized and efficient tumor ablation while minimizing damage to surrounding healthy tissues [57,58].

#### 3.1.3 Electrical response

The electro-responsive strategy induces changes in the structure or charge state of the material through the application of electrical stimuli to achieve the controlled release of drugs and the modulation of cellular behavior, thereby accurately remodeling the tissue repair microenvironment [17]. For example, electroactive materials can mimic the piezoelectric properties of dentin or respond to exogenous electric fields to activate odontogenic signaling pathways, such as the cAMP-PKA [59] and ERK [60] pathways, in dental pulp stem cells (DPSCs). These effects contribute to the formation of pulp-like tissue, providing a promising strategy for dental pulp regeneration therapy.

#### 3.1.4 Magnetic response

External magnetic fields can penetrate human tissues and remotely manipulate magnetic nanomaterials (e.g., Fe_3_O_4_ nanoparticles) [61,62] for targeted delivery or magneto-thermal-responsive release guided by a magnetic field, which can effectively alleviate the inflammatory microenvironment and promote tissue regeneration [25,61].

#### 3.1.5 Ultrasound response

Ultrasound stimuli can elicit the targeted release of drugs by penetrating tissues and destroying specific material structures to build an efficient local drug delivery system. The underlying mechanisms include mechanical, thermal, and cavitation effects. Through these effects, ultrasound stimuli can activate sonosensitizers to generate ROS in the presence of H_2_O and O_2_ (Fig. 2B). These ROS exert antibacterial or antitumor effects [25] through oxidative stress, a process known as sonodynamic therapy (SDT) [63]. In contrast, PDT relies on light in the visible or NIR spectrum, which has limited tissue penetration. Therefore, PDT is more suitable for treating superficial infections, such as those on the tooth surface, exposed root surfaces, and oral mucosa [64]. Compared to PDT, ultrasound can penetrate tissues deeper; moreover, ultrasound equipment is highly available in dentistry, making SDT a promising approach for treating deep-seated oral infections or lesions [65].

Although physical stimulus-responsive nanomaterials offer precise spatiotemporal control, their clinical use may be limited by the need for device assistance or shallow penetration of tissues. Additionally, such approaches may pose risks of collateral damage to healthy tissues and generally exhibit lower specificity compared to chemical-responsive systems that passively target pathological microenvironmental cues [66].

### 3.2 Chemical stimulus response

Chemical stimulus-responsive nanomaterials operate by sensing endogenous biochemical changes in the pathological microenvironment [66], such as variations in pH and redox potential. These internal signals are hallmarks of oral diseases, including inflammation, infection, and tissue degeneration, and serve as natural triggers for targeted activation. By integrating chemical sensing elements into their design, smart nanomaterials can undergo controlled degradation, drug release, or structural transformation in response to specific pathological conditions. This autonomous, microenvironment-driven behavior improves therapeutic specificity and reduces off-target effects, making chemical responsiveness a powerful strategy for precision medicine in oral tissue regeneration.

#### 3.2.1 pH response

Another important biochemical indicator in human systems is pH, and changes in pH are key regulators in material design and precision medicine [25]. Diseases such as oral inflammation, caries, and oral cancer are often accompanied by local acidification (e.g., periodontitis and tumor tissue pH < 6.5, caries lesions pH < 5.0). Acid-responsive strategies induce the release of drugs mainly through the introduction of acid-sensitive chemical bonds (e.g., hydrazones, imines, esters, and amides) and ionizable groups (e.g., carboxyl groups and amino groups) to achieve structural disassembly or protonation modulation in acidic environments [25,67]. Additionally, some pH-responsive systems increase local target adhesion along with structural rearrangement by linking with the local pH of the lesion to increase therapeutic efficiency and local residence time [68]. For example, metal-organic frameworks (MOFs) depend on the acid sensitivity of the metal-ligand–ligand bond, and the structure collapses under acidic conditions [69]. Natural polyphenols rich in catechol groups can form dynamic complex structures with metal ions, and complex dissociation occurs under acidic conditions and the influence of protonation, which achieves both structural response and drug release [70].

#### 3.2.2 Redox response

Redox-responsive nanomaterials can sense changes in the redox state *in vivo* and perform functional release or bioregulation to reestablish cellular redox homeostasis. Among numerous redox-responsive materials, nanozymes are a key tool for regulating the local oxidative environment as they perform enzyme-like activity, have high catalytic efficiency, and are stable and inexpensive [71]. Moderate levels of ROS can serve as therapeutic “weapons” for antibacterial, antitumor, or pigment-degrading applications. For example, some nanomaterials that exhibit peroxidase (POD)-like [72], oxidase-like, and glucose oxidase (GOx)-like [73] activities can catalyze the conversion of H_2_O_2_ into highly reactive •OH and O_2_^•–^ (Fig. 2C), thereby enhancing oxidative stress to exert antibacterial effects and helping eliminate infection sites [74,75]. These materials can also promote tooth whitening through the oxidation of pigment precursors [76,77] or induce apoptosis and ferroptosis in ROS-sensitive cancer cells for treating oral cancer [78]. This catalytic process forms the basis of chemodynamic therapy (CDT), an emerging therapeutic strategy that leverages Fenton or Fenton-like reactions to generate cytotoxic ROS *in situ*, particularly in the infection microenvironment [16].

For scavenging ROS, nanozymes with superoxide dismutase (SOD)-like activity can catalyze the conversion of O_2_^•–^ into H_2_O_2_ and O_2_, whereas those with catalase (CAT)-like activity further decompose H_2_O_2_ into H_2_O and O_2_. These antioxidant nanozymes effectively decrease ROS levels, mitigate oxidative stress, suppress inflammatory responses, protect cellular functions, and accelerate tissue repair [40]. Redox modulation is not limited to antioxidant nanozymes. Certain materials can neutralize ROS through chemical interactions, such as phenolic groups, or capture highly reactive species such as •OH and O_2_^•–^ via electron-rich nanostructures, thereby preventing oxidative damage to cells and tissues (Fig. 2D). Antioxidant therapy aims to fine-tune ROS levels in a therapeutically beneficial range instead of eliminating them [79]. The generation and scavenging mechanisms of reactive oxygen free radicals are summarized in Fig. 2 [16]. These redox strategies together provide a promising foundation for applications in inflammation control, cancer treatment, and tissue regeneration.

### 3.3 Biological stimulus response

Biological response strategies represent important advancements in smart nanomaterial design. These systems can sense and respond to endogenous biological signals through highly specific molecular recognition and active targeting, triggering conformational changes or signal transduction in response to cues such as the overexpression of enzymes [80] and receptor-ligand interactions [81], in diseased oral tissues. Among all stimulus-responsive approaches, biological strategies exhibit the highest targeting specificity. Compared to physical and chemical stimuli, this shift from broad environmental triggers to finely tuned molecular recognition represents a deeper integration with the biological microenvironment.

#### 3.3.1 Enzyme response

Enzyme-responsive strategies use specific enzymes secreted by pathogens during infection or tissue repair to catalyze the degradation of polymers embedded in the material, thereby inducing structural rupture of the material or disintegration of the drug-carrying system for selective release of the drug [80]. Different types of enzymes recognize and degrade specific substrates, conferring a high degree of sensitivity to microenvironmental changes in the material [17].

#### 3.3.2 Receptor-mediated response

Specific receptors on the surface of cells can mediate the target recognition of nanomaterials. For example, nanoparticles modified with RGD peptides on the surface can bind to osteoblasts via integrins and increase the osteoinductive capacity of the materials [81].

#### 3.3.3 RNA interference

RNA interference technology provides novel regulatory strategies for tissue repair. By loading small interfering RNAs or microRNAs using nanocarriers, gene expression can be precisely regulated at the post-transcriptional level, which in turn affects various cellular behaviors [82].

However, biological stimulus-responsive systems also present certain limitations. Enzyme expression levels can vary between individuals and fluctuate over time, making response kinetics difficult to predict. Additionally, the complex bio-recognition motifs employed require careful validation to avoid unintended immune reactions. The fabrication processes are often more intricate, and the response times may be relatively slow. In practice, purely biological triggers are frequently combined with physical or chemical cues to enhance specificity and achieve more precise control.

While physical, chemical, and biological stimuli each offer unique advantages in triggering specific functional responses, the complexity of oral pathological microenvironments often involves multiple overlapping cues. Depending on a single stimulus may, therefore, be insufficient to achieve optimal therapeutic outcomes. Hence, researchers have focused on multi-responsive nanomaterials that integrate two or more stimulus-responsive elements [17].

### 3.4 Multi-modal composite response

Owing to the complexity of the oral microenvironment, a single stimulus response may have difficulty meeting the needs of practical applications. Smart materials based on multi-modal composite response mechanisms have become important in designing tissue regeneration materials. These materials can sense multiple internal and external stimuli simultaneously and achieve more precise release and regulation of biological functions in complex microenvironments. For example, Li et al. designed a dual pH/UV-responsive hydrogel wound dressing that not only facilitates antibacterial and regenerative activity via pH-triggered release of tannic acid (TA) and adenine in acidic environments but also reduces skin adhesion upon exposure to UV light through the Fe^3+^-Fe^2+^ transition, decreasing secondary injury during removal [70]. Similarly, Xu et al. reported a pH/NIR-responsive photothermal antibacterial “warm paste” designed to target cariogenic biofilms. This system, known as FePAgPG, was developed by sequentially modifying Fe_3_O_4_ nanoparticles with polydopamine (PDA), silver, another PDA layer, and glycol chitosan. In the acidic microenvironment, FePAgPG nanoparticles selectively target pathogenic bacteria through their pH-responsive activity. Exposure to NIR light causes the nanoparticles to exhibit effective bactericidal effects via a silver-enhanced PTT strategy. The magnetic properties of Fe_3_O_4_ facilitate the external removal of the nanoparticles, thereby mitigating the toxicity associated with residual nanomaterials. This approach serves as a promising reference for the development of photothermal nanomaterials that target open wound infections beyond the oral cavity [83]. These stage-specific response mechanisms highlight the higher therapeutic precision and biosafety offered by multi-modal strategies-capabilities that are absent in single-stimulus platforms. Overall, the synergistic mechanisms underlying multi-modal responsiveness represent a forward-looking trend in smart-responsive material design, characterized by integrated multi-stimuli sensitivity and greater functional versatility.

Smart-responsive nanomaterials for oral applications have versatile functionality because of their tunable surface chemistry and hierarchical structures, which facilitate precise responses to physical, chemical, and biological stimuli. While single-stimulus systems provide targeted effects, they often have restricted functionality in the complex oral microenvironment. In contrast, multi-modal platforms integrate multiple triggers to increase spatiotemporal precision and achieve synergistic therapeutic outcomes, establishing a new paradigm for intelligent nanomaterial design.

## 4 The application of a smart response to microenvironment nanomaterials in oral tissue regeneration

### 4.1 Tooth tissue regeneration

Tooth tissues are composed of enamel, dentin, cementum, and pulp, each with a unique structure and function. The enamel is a highly mineralized hard tissue that covers the surface of the crown [84]. It has an extremely high content of inorganic components and is the first site to be attacked by dental caries. Once damaged, it cannot regenerate spontaneously. Dentin is the main structure of the tooth body. It is rich in organic matrix and collagen, supports the enamel, protects the pulp, and has some regenerative capacity, especially under the action of odontoblasts, which can facilitate repair. The dental pulp is the only soft tissue in the tooth. It contains abundant blood vessels and nerves, is responsible for dentin formation, and participates in functions such as nutrition supply, sensory conduction, and immune defense [82]. The cementum is a bone-like tissue that covers the surface of the tooth root. It is similar in composition to bone tissue but with lower hardness, and it is the main tissue that connects the tooth to the periodontal supporting structures.

Dental defects are commonly caused by traumatic injury, dental caries, developmental anomalies, or chronic inflammation [7,85]. They are frequently accompanied by disturbances in the local microenvironment, such as pH fluctuations, an increase in oxidative stress, bacterial infection, and persistent inflammatory responses. These changes interfere with self-repair processes and exacerbate damage, particularly during the progression of dental caries and pulpitis.

Dental caries is a chronic, progressive disease that destroys the hard tissues of teeth and is caused by multiple factors, such as the host, microorganisms, the oral environment, and time [86]. It mainly occurs due to the fermentation of dietary carbohydrates by dental plaque on the tooth surface, which produces acid that lowers the local pH to 4.5–5.5, inducing demineralization at the biofilm–enamel interface [36,87]. Further progression can cause irreversible damage to dental tissues. The commonly used treatments include the application of fluoride, mouthwash, and artificial fillings. However, artificial fillings fail to reconstruct the structure and function of enamel and are prone to microleakage and secondary caries [36,88]. The long-term use of chlorhexidine (CHX) mouthwashes can cause staining, microbial imbalance, and increased antibiotic resistance; moreover, they cannot remineralize. Fluorides can partially inhibit demineralization but may lead to fluorosis or disrupt the microbial balance [83,89], and their overall therapeutic effects are limited. When treatment is not administered on time, bacteria can invade deeper parts of the tooth, leading to dentin hypersensitivity, pulpitis, and even periapical periodontitis. In severe cases, it can cause tooth loss.

Pulpitis and periapical periodontitis are commonly treated with root canal therapy, which completely removes the infected pulp tissue and dentin in the root canal and tightly seals the root canal system with biologically inert materials [90]. However, this treatment method cannot promote the regeneration of dental tissues. Moreover, complex root canal anatomy, different degrees of inflammation, and individual patient differences may lead to unpredictable pulp reactions, resulting in permanent pulp inactivation, secondary infection, tooth fracture, or even tooth loss [59,91]. The regeneration of dental tissues is an important research direction in dentistry. Smart-responsive nanomaterials that can dynamically monitor and respond to the above-mentioned pathological states can promote the regeneration of dental tissues in several ways (Table 1).

**Table 1:** Representative materials and functional mechanisms of smart response nanomaterials in dental tissue regeneration.

Stimuli-responsive Type	Synthesis Strategy	Nanoplatform	Target Cells/Tissues	Regeneration Function	Ref.
PDT/US	Hydrothermal method	Sr-ZnO@PDA	Enamel, Dentin, *S. mutans.*	ROS generation, Prevent caries, Improve tooth staining, Promote the remineralization.	[92]
pH/PTT	Solvothermal method	Mg-MOF@PDA@CaP (MPC) composite foam brace	*S. mutans,* *S. sobrinus,* *A. viscosus,* Enamel.	Destroy the biofilm, Optimize the oral microenvironment, Tooth hard tissue self-healing.	[69]
PTT/Thermo-responsive	Self-assembly	BP@CP5 hydrogel	*S. mutans,* *S. sanguinis,* Enamel.	Wet adhension, Antibacterial, Promote the remineralization.	[93]
ROS/pH	Coordination-reduction combined biomineralization strategy	GLA/GS hydrogel	Acidic plaque biofilm, Enamel.	Antibacterial, Inhibit biofilms, Promote the remineralization.	[94]
ROS	Two-step ion exchange liquid-phase stripping method	VMT/ACP/Dextran nanosheets	Dentin, Dental plaque.	Inhibit cariogenic bacterial adhesion and biofilm formation, Promote dentin tubules occlusion.	[95]
pH	Coordination interaction	EGCG-ACP nanocomposite	Enamel, cariogenic biofilm.	Antibacterial, Promote the remineralization.	[96]
pH	Thiol-ene click chemistry	Multifunctional peptide/pVEGF gene complex	Dental pulp (full-length tooth root)	Angiogenesis, Promote pulp regeneration.	[97]
Thermo-responsive		CH hydrogel with AZI	*E. faecalis,* Dental pulp, Apical tissues.	Antibacterial, Improve mineralized tissue formation and vascularization.	[46]
ROS		DFSC-sEVs-loaded SA-RhB hydrogel	Dental pulp (inflamed DPSCs)	Antioxidant, Restore mitochondrial function, Promote pulp regeneration.	[98]
Electrical-responsive		KNN and PKNN nanoparticles	Dental pulp (DPSCs)	Induce odontogenic differentiation of DPSCs, Promote pulp regeneration	[59]
Electrical-responsive		P(VDF-TrFE) films	Dental pulp (DPSCs), Dentin.	Induce odontogenic differentiation of DPSCs, Secrete reparative dentin, Odontogenesis.	[60]
PTT	Hydrothermal method	SrCuSi_4_O_10_(SC)/GelMA hydrogel	*L. casei,* *S. mutans.*	Antibacterial, Angiogenesis, Odontogenesis.	[51]

#### 4.1.1 Dental caries and remineralization

Dental caries and remineralization reveal the delicate balance between the destruction and self-repair of hard dental tissues. Owing to their lack of dynamic responsiveness and ability to synergize antibacterial and remineralization functions, traditional remineralization materials struggle to effectively reverse the imbalance between demineralization and remineralization. Smart-responsive nanomaterials can target pathological signals based on changes in the oral environment. They can perform precise antibacterial activity and biofilm elimination while releasing remineralizing ions on demand. These materials also increase retention efficacy in the dynamic oral environment through biomimetic adhesion strategies, thus providing a new pathway to improve dental tissue regeneration.

**Fig. 3:**
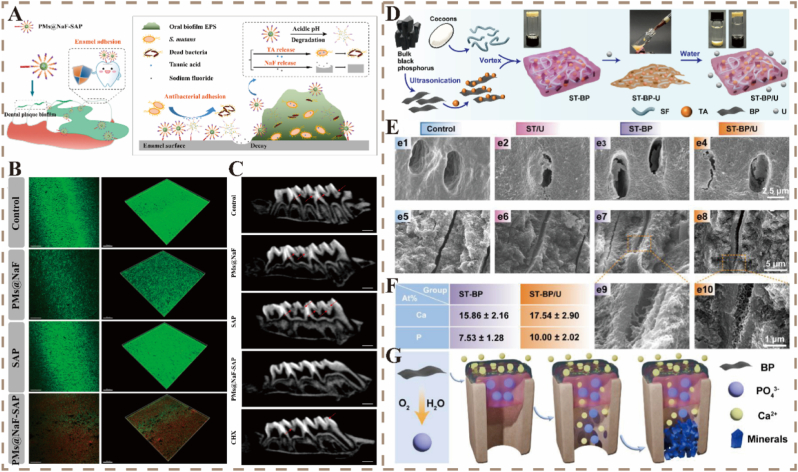
A) Schematic of PMs@NaF-SAP topical application on dental plaque biofilms and its mechanism for caries prevention and enamel repair. B) Confocal laser scanning microscopy images showing anti-*S. mutans* biofilm effects under various treatments. C) 2D sagittal micro-CT images of maxillary molars [99]. Reproduced with permission from Ref. [99] Copyright 2023, Elsevier. D) Synthetic route of ST-BP-U and its transformation upon water exposure. E) SEM images and F) EDS analysis of bovine root dentin under different treatments. G) Schematic illustration of the remineralization mechanism. [101]. Reproduced with permission from Ref. [101] Copyright 2024, Elsevier.

The low-pH environment caused by acid production from the metabolism of cariogenic bacteria in the oral microenvironment is the main force driving the occurrence and development of dental caries. Nanomaterials based on the pH-responsive nature of the carious microenvironment have become a research hotspot. Xu et al. combined bacterial responsiveness with biomimetic adhesion to develop a pH-responsive multidrug delivery system known as PMs@NaF-SAP (Fig. 3A). This system, which is conjugated with salivary-acquired peptide (SAP), can specifically adhere to dental enamel, thereby avoiding the buffering effect of saliva in the oral cavity [99,100]. The system can also recognize cariogenic conditions and cleave boronate ester bonds at pH ≤ 5.0, releasing TA to inhibit cariogenic bacteria and sodium fluoride (NaF) as a remineralizing agent to promote faster remineralization and better hydroxyapatite (HA) crystal growth. Compared to PMs@NaF, SAP alone, or the control, PMs@NaF-SAP has a significantly greater inhibitory effect on *S. mutans* adhesion (Fig. 3B). Sagittal section images revealed that enamel treated with PMs@NaF-SAP presented fewer demineralization sites (Fig. 3C). This treatment leads to targeted inhibition of pathogens in acidic environments and repairs the microstructure and mechanical properties of demineralized teeth, avoiding the effects of broad-spectrum antibiotics on the oral microbiota [99]. In that study, polymer micelle nanomaterials with a classic spherical core-shell structure were used as a drug delivery system to protect drugs from pH degradation in the harsh ecological niche of biofilms while using a unique microenvironment for smart-responsive drug release. The CP-SAP system, which is based on the interrupting dental caries theory and combines PSs with SAP, also achieves a comprehensive effect of sustained adhesion, photodynamic antibacterial activity, and remineralization. Its delivery system includes peptide dendrimer nanogels, which, however, do not possess environment-responsive features [100].

The continuous flow of fluids and mechanical activity in the oral cavity make it difficult for drug delivery platforms to maintain stable high-concentration release, especially in the treatment of root caries, where the persistent production of gingival crevicular fluid increases the difficulty of fluid isolation. Therefore, a drug-release platform that can firmly adhere to irregular dental surface structures in a moist environment needs to be developed for the long-term management of dental caries. By studying the slime of hagfish, Zhu et al. developed a water-responsive fluid-to-hydrogel transition system. They combined black phosphorene (BP) with TA and silk fibroin (SF) to form a gelatinous ST-BP, which was then mixed with urea (U) to generate a fluidic ST-BP-U. U disrupts the hydrogen bonds in the SF-TA complex, making the system fluid and injectable, facilitating its penetration into the lesion area and filling the dentinal tubules. Upon contact with saliva or gingival crevicular fluid, U diffuses away, allowing the re-establishment of the hydrogen bonds and the BP cross-linking network, thereby forming the ST-BP/U hydrogel (Fig. 3D). This hydrogel achieves mechanical interlocking and robust adhesion with the irregular root surface, effectively sealing the dentinal tubules and preventing further demineralization. In the ST-BP/U group, the dentinal tubules were almost completely occluded in cross-sectional view (Fig. 3E), accompanied by an increase in calcium (Ca) and phosphorus (P) contents (Fig. 3F) [101]. Similarly, the STAG hydrogel rapidly solidified after injection by disrupting hydrogen bonds with guanidine hydrochloride (Fig. 3G). The release of Ca^2+^ from amorphous calcium phosphate nanoparticles enhances their adhesion and remineralization capabilities [102]. This mechanism of hydrogen bond disruption and reformation imparts fluidity and wet adhesion properties to the hydrogel. This helps the hydrogel achieve efficient adhesion and remineralization on the irregular root surface in the complex oral environment.

The intelligent system mentioned above can significantly decrease the incidence of dental caries and repair demineralized tissues in animal models. Its therapeutic efficacy is substantially greater than that of the gold standard oral antibacterial agent CHX (Fig. 3C), which has higher biocompatibility [99,100]. Most of these systems maintain the balance of the oral microbiota [94,99], avoiding the ecological disruption caused by traditional antibacterial agents. Its main advantage includes achieving precise microenvironment regulation through multiple synergistic mechanisms and biomimetic design. However, clinical translation needs to address challenges such as the long-term stability of materials, operability under complex stimuli, and dynamic adhesive durability.

#### 4.1.2 Pulpitis and pulp regeneration

Dentin and pulp originate from dental papilla tissue during the embryonic period and together form the dentin-pulp complex, which is highly coordinated in structure and function. Maintaining integrity is essential for preserving tooth vitality and achieving tissue regeneration [90], a process that relies on the differentiation of DPSCs into odontoblast-like cells [103]. Therefore, constructing a microenvironment that favors their proliferation, migration, and directed differentiation is necessary for research on tissue regeneration. For early pulp exposure or superficially infected vital pulp, pulp capping therapy can be administered to isolate external stimuli, control infectious inflammation, and promote the repair of the pulp-dentin complex. Traditional materials induce reparative dentin but lack responsiveness to pathological signals, often resulting in poor preservation of pulp or limited tissue function. In contrast, smart-responsive nanomaterials sense microenvironmental cues to precisely control the release of therapeutic agents, promoting the odontogenic differentiation of DPSCs, angiogenesis, antibacterial activity, and immune modulation, ultimately improving the outcomes of pulp treatment.

**Fig. 4:**
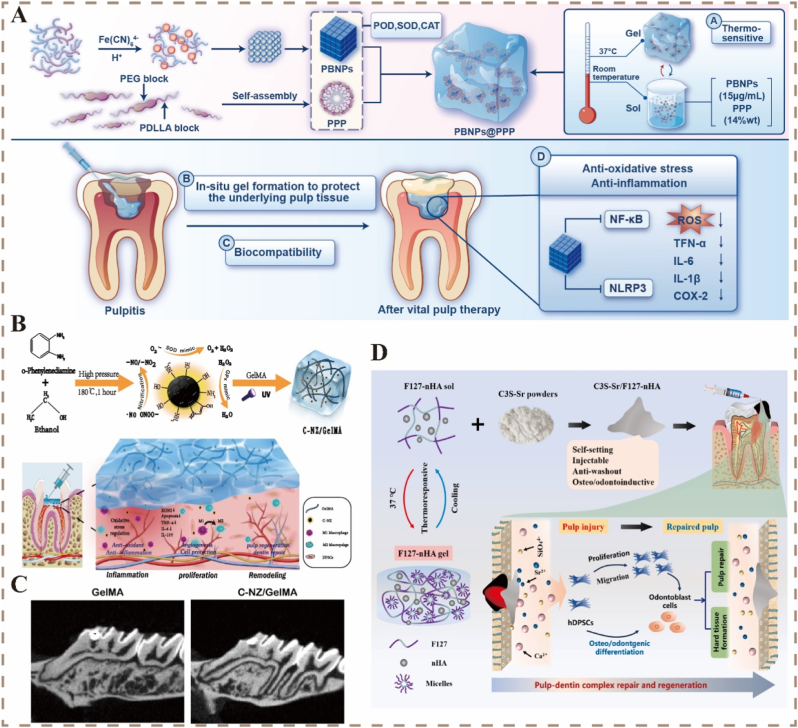
A) Schematic of PBNPs@PPP structure and its therapeutic process for vital pulp therapy [50]. Reproduced with permission from Ref. [50] Copyright 2025, Elsevier. B) Illustration of C-NZ/GelMA hydrogel synthesis and its application in pulpitis treatment. C) Micro-CT images showing reparative dentin formation at defect sites [71]. Reproduced with permission from Ref. [71] Copyright 2024, Springer. D) Mechanism of C3S-Sr/F127-nHA in promoting pulp-dentin complex repair and regeneration [49]. Reproduced with permission from Ref. [49] Copyright 2025, Elsevier.

The local accumulation of inflammatory mediators and oxidative stress is are major factors that interfere with the pulp regeneration process. By scavenging ROS or regulating immune responses, smart-responsive materials can reshape a microenvironment conducive to tissue repair. Researchers have developed various antioxidant delivery systems to promote the recruitment of stem cells and their differentiation into the dentin-pulp complex [103]. Prussian blue nanoparticles (PBNPs) can effectively scavenge ROS by mimicking the activities of SOD, CAT, and POD [104]. They can also regulate the secretion of ROS-mediated inflammatory factors, resulting in excellent antioxidant and anti-inflammatory capabilities. These properties have led to their widespread application in the treatment of inflammatory diseases and dental pulp regeneration. Fig. 4A illustrates the design, structural components, and therapeutic mechanism of a novel pulpitis treatment strategy in which a triblock copolymer (PPP) is used as a biodegradable scaffold to deliver PBNPs (PBNPs@PPP) [50]. Owing to the reversible Ce^3+^/Ce^4+^ transformation on their surface, cerium oxide nanoparticles (CNPs) can neutralize O_2_**^•^**^–^ and H_2_O_2_ in oxidative stress responses, thereby alleviating local inflammation and promoting the osteogenic/dentin differentiation of hDPSCs [[105], [106], [107]]. The carbon dot nanozyme/gelatin methacryloyl composite hydrogel (C-NZ/GelMA) (Fig. 4B) developed by Zhang et al. can efficiently scavenge ROS and nitrogen species, thereby alleviating damage caused by oxidative stress in pulpitis and maintaining redox homeostasis [71]. This system can also induce the polarization of M2 macrophages, improve the immune microenvironment, and promote pulp regeneration and dentin repair (Fig. 4C). It has great potential for clinical application [37,71].

Thermo-responsive materials, owing to their excellent shape adaptability and tissue compatibility, are highly promising in the regeneration of dental pulp. For example, Wu et al. incorporated a thermoreversible Pluronic F127 hydrogel and nano-HA (nHA) into a tricalcium silicate (C3S) cement system doped with SrCO_3_ (C3S-Sr/F127-nHA), thereby improving the injectability and anti-collapse properties of the material. Adding nHA enhanced the hydration and mineralization capabilities of C3S. *In vivo*, it induced the formation of a uniform dentin bridge, significantly promoting the regeneration of the pulp-dentin complex (Fig. 4D) [49]. To enhance bioactivity and regenerative efficiency, Wang et al. synthesized a thermosensitive hydrogel based on hydroxypropyl chitin (HPCH) and chitin whiskers (CWs) and encapsulated exosomes derived from hDPSCs to form an HPCH/CW/Exo hydrogel [108]. This hydrogel can deliver exosomes to precise locations, thereby enhancing odontogenic and angiogenic capabilities and effectively promoting the formation of pulpoid tissue, providing a regenerative alternative to traditional root canal therapy.

The regeneration of the dentin-pulp complex depends on the synergistic regulation of multiple factors and stages. The development of multidimensional smart-responsive systems, such as redox, temperature, and electric field systems, not only increases the survival and differentiation efficiency of stem cells but also improves the precision and clinical feasibility of regenerative therapy.

### 4.2 Periodontal tissue regeneration

The periodontium is a critical supporting structure that anchors teeth in the alveolar bone and comprises four main components: the gingiva, PDL, cementum, and alveolar bone [109]. The gingiva is a soft tissue barrier covering the cervical region of the tooth and the alveolar ridge. It serves as the first line of defense against microbial invasion. The PDL, located between the cementum and alveolar bone, is rich in blood vessels, nerve fibers, and fibroblasts and plays key roles in mechanical support, sensory perception, and nutrient exchange. The cementum is a mineralized tissue covering the tooth root that lacks vascularization and innervation but provides a critical anchoring surface for the PDL. The alveolar bone offers structural support for the teeth and is highly plastic, undergoing continuous remodeling in response to masticatory forces. These components together function synergistically to maintain the integrity and stability of tooth attachment.

Periapical periodontitis, periodontal disease, dental trauma, and maxillofacial tumors frequently result in defects in the dentition and alveolar bone [110]. Periodontal disease is a chronic bacterial infection characterized by progressive alveolar bone resorption, leading to tooth mobility or even tooth loss, thereby severely compromising oral function and systemic health [111]. Its pathogenesis involves bacterial invasion, chronic inflammation, oxidative stress [112], and disruption of the local microenvironment. During the inflammatory response, immune cells release ROS to eliminate pathogens; however, high levels of ROS increase the production of proinflammatory mediators, exacerbating inflammation and establishing a deleterious “ROS-inflammation” feedback loop. This vicious cycle aggravates tissue damage and inhibits the proliferation and differentiation of periodontal ligament stem cells (PDLSCs), thus impairing the repair and regeneration of periodontal tissues [113]. The goal of periodontal therapy is to restore the soft and hard tissues of the periodontal complex, with alveolar bone regeneration serving as a critical component [114,115].

The main objective of conventional periodontal treatments, such as scaling, root planning, flap surgery, and bone grafting, is to remove dental plaque and control infection. However, these approaches are often invasive and associated with long treatment durations, incomplete debridement, and limited regenerative outcomes [116], making it difficult to achieve full structural and functional restoration of the periodontium [117]. Exogenous graft materials may also trigger immune rejection or fibrotic responses. Although widely used bone substitutes and barrier membranes exhibit some degree of bioactivity, their stability and regenerative performance are often compromised in inflamed environments. The dynamic and complex nature of the oral cavity, including variations in salivary flow, chemical composition, and temperature, further accelerates drug loss [68], whereas the irregular anatomical architecture of periodontal pockets presents additional challenges for effective delivery and retention.

Smart-responsive nanomaterials not only eliminate pathogenic bacteria and biofilms but also modulate oxidative stress, alleviate inflammation, optimize the periodontal microenvironment, inhibit alveolar bone resorption, and promote bone regeneration, thereby offering comprehensive support for the repair of soft and hard periodontal tissues [118]. Among the main cellular contributors to periodontal regeneration are PDLSCs, which originate from the PDL and serve as essential seed cells during regeneration [109]. An ideal regenerative material should simultaneously support the immunomodulatory microenvironment and direct the fate of PDLSCs [114]. In this section, we discussed the major pathological mechanisms underlying periodontitis and highlighted recent advancements in smart-responsive nanomaterials for periodontal regeneration, particularly their functional strategies in terms of antibacterial action, immunomodulation, antioxidation, glycemic regulation, angiogenesis, and osteogenesis (Table 2). These strategies provide new insights and material foundations for future periodontal regenerative therapy.

**Table 2:** Representative materials and functional mechanisms of smart-responsive nanomaterials in periodontal regeneration.

Stimuli-responsive Type	Synthesis Strategy	Nanoplatform	Target Cells/Tissues	Regeneration Function	Ref.
PTT	Microfluidic encapsulation, Surface modification	GM@Fe-Cur/PDA/MH microspheres	Macrophages, Alveolar bone	Antibacterial, Antioxidant, Anti-inflammatory, Bone regeneration.	[119]
PDT/PTT	Liposome encapsulation	ICG-rapamycin nanoparticles	Macrophages, Bacterial biofilms	Antibacterial, ROS generation, Immunomodulation, Biofilm inhibition.	[34]
PTT/NO	Room-temperature synthesis	SPBzyme nanoparticles	Periodontal tissues, Bacterial biofilms	Antibacterial, Antioxidant, Anti-inflammatory.	[120]
pH	Alkali-assisted carbonization process	RSV-CPDs@QCS/OD (RCQD) hydrogel	Periodontal tissues, Bacterial biofilms	Antibacterial, Antioxidant, Immunomodulation, Anti-inflammatory, Tissue regeneration.	[121]
NO	Sol-gel synthesis, Surface modification	MBG@L-Arg nanoparticles	Macrophages, MC3T3-E1 cells, hPDLSCs	ROS scavenging, Antioxidant, Anti-inflammatory, Osteogenesis.	[122]
Thermo-responsive	One-pot method	SFD/CS/ZIF-8@QCT hydrogel	PDLSCs, Macrophages, Alveolar bone	Antibacterial, Antioxidant, Immunomodulation, Pro-recruitment, Osteogenesis, Angiogenesis.	[123]
Wet	One-step solvent volatilization concentration method	PolyLA-GelMA patch	Periodontal tissues	Antibacterial, ROS scavenging, Blood compatibility, Anti-inflammatory.	[124]
Piezoelectric	Sol-vothermal reaction, Thermal calcination	GelMA + t-BTO hydrogel	PDLSCs, Macrophages, Periodontal tissue	Immunomodulation, Anti-inflammatory, Osteogenesis, Energy metabolism modulation.	[125]
ATP	Solvent thermal method	Mg/Zn-MOF	Immune cells, Periodontal tissue	Pyroptosis inhibition, Immunomodulation, Anti-inflammatory, Collagen preservation, Bone loss prevention.	[126]
pH	Thiol-ene click reaction	NG-TCS-DFO nanogel	*P. gingivalis,* HUVECs, Periodontal tissue	Antibacterial, Osteogenesis, Angiogenesis, Periodontitis healing.	[127]
Enzyme		TM/BHT/CuTA hydrogel	Macrophages, Periodontal tissue	Antibacterial, Anti-biofilm, ROS scavenging, Anti-inflammatory, Osteogenesis, Tissue regeneration.	[128]
ROS	Hydrothermal coprecipitation reaction	CoO–Ir	Periodontal tissue, Osteoblasts	ROS scavenging, Anti-inflammatory, Osteogenesis.	[129]
PTT/PDT	Water-induced self-assembly method	VP/Ti_3_C_2_ VDW heterojunction	*S. aureus,* *E. coli,* Periodontal soft and hard tissues	Antibacterial, ROS generation, Anti-inflammatory.	[130]
SDT	Hydrothermal method	Zn(OH)F/CaF_2_ heterojunction	*P. gingivalis,* *S. aureus,* Periodontal tissue	Antibacterial, Osteogenesis, Bone loss prevention.	[131]
PDT	Hydrothermal method	I-MoO_3-x_ nanobelts	*S. aureus,* *E. coli,*	Antibacterial	[132]
PTT	Ammonia etching	Au@CeO_2_-DMF nanozyme	Periodontal tissues	Antioxidant, Mitochondrial maintenance, Immunomodulation.	[133]
Thermo-responsive	Emulsified solvent evaporation method	M-CNPs@Gel nanodecoy system	Monocyte, Macrophages	ROS scavenging, Immunomodulation.	[134]
Glucose-responsive	One-pot hydrothermal method	Au/Pt NCs@GOX clusterzyme	Periodontal tissue, *F. nucleatum* biofilm	Inhibits biofilm formation, Anti-inflammatory, Tissue regeneration.	[135]
PTT-/Glucose-/Thermo-/pH responsive	Chemical coprecipitation method	AgZ@Au/PLEL hydrogel	Periodontal pocket, Bacteria, BMSCs	Antibacterial, Promote glucose decomposition, Osteogenesis.	[136]
ROS	Solvent evaporation method	RSV@DTPF nanoparticles	Macrophages, Osteoblasts, Osteoclasts	ROS scavenging, Immunomodulation, Inhibits Osteoclastogenesis, Osteogenesis.	[137]
PTT		RSV-Au@H hydrogel	Osteoblasts, Periodontal tissue	ROS scavenging, Mitochondrial maintenance, Osteogenesis.	[138]
ROS	Thiol-ene click chemistry	G8–0 hydrogel	BMSCs, Periodontal tissue	ROS scavenging, Restores GSH/GSSG balance, Osteogenesis.	[139]
PDT	One-pot method	Pt@PCN-222	Anaerobic bacteria, Inflamed tissues	Anti-infection, Anti-inflammatory.	[140]
PTT/CDT		MN-SF/CuS microneedles	Periodontal pocket tissues, Bacteria	ROS generation, Antibacterial, Tissue regeneration.	[141]
ROS	*In situ* reduction and deposition approach	MnO_2_@UiO-66(Ce)	PDLSCs, Alveolar bone	Antioxidant, Restores mitochondrial homeostasis, Anti-inflammatory, Osteogenesis.	[142]
NIR-IIb light/pH	Thermal decomposition method, Surface modification	DMUP (Mn nanozyme + Pae + UBI-DCNPs)	Periodontal tissue, Macrophages, Alveolar bone	Bacterial imaging, Anti-anaerobic bacteria, ROS scavenging, Anti-inflammatory, Promoting bone regeneration.	[143]
PTT/PCAT/ROS	Seed-mediated growth method	GNRs@CeO_2_@PDS Janus heterostructure + persulfate	Periodontal pathogens, Inflamed periodontal tissue, *F. nucleatum*, *P. gingivalis*	Antibacterial, Eliminate biofilms, ROS generation, Anti-inflammatory, Bone preservation.	[144]
ROS		MIL-47(V)-F (MVF) nanozyme	PDLSCs, Inflammatory periodontal tissues	ROS scavenging, Anti-inflammatory, Immunomodulation, Promote periodontal regeneration.	[145]
PTT/CDT/ROS		GDY-Fe@HA-DA hydrogel	*P. gingivalis,* *S. aureus,* *E. coli,* Periodontal tissues, Infected wounds	Antibacterial, Anti-inflammatory, Promote wound healing, Tissue regeneration.	[146]
PDT/ROS	Washing centrifugation	TA-loaded PCOF	Periodontal infection sites	Antibacterial, ROS generation.	[147]
ROS	Self-assembly	FeSN nanozyme	Pathogenic oral biofilms, Periodontium	Antibacterial, Eliminate biofilms.	[148]
ROS		Pt@ZIF-8/ALN-ac/Gel hydrogel	Inflammatory periodontium, *P. gingivalis,* hPDLSCs	Antibacterial, ROS scavenging, Osteogenesis, Reduce osteoclast activation.	[149]
PTT/ROS	Electrostatic extrusion	Ag-TiO_2-x_@alginate microspheres	Periodontal pathogens, *P. gingivalis*, *S. gordonii*	Antibacterial, ROS generation.	[150]
pH	Self-assembly	Au@MPN-BMP2	BMSCs, Inflammatory alveolar tissue	Antibacterial, ROS scavenging, Osteogenesis.	[151]
PTT	Electrostatic interaction	Fe_3_O_4_/ZnO/EPL nanoparticles (FZE NPs)	Oral biofilms, *P. gingivalis,* Periodontal tissue	Antibacterial, Eliminate biofilms.	[152]
Infection-sensitive	Layer-by-layer assembly	SPION/PLGA scaffold	*P. gingivalis,* Macrophages, BMSCs, Periodontal tissue	Antibacterial adhesion, Immunomodulation, Periodontal regeneration.	[61]
PTT/Thermo-/ROS responsive	Electrostatic and hydrogen bonding interactions	PF-127/HAMA/M@S (PH/M@S) hydrogel	Periodontal pocket, Inflamed tissue	Antibacterial, ROS scavenging, Anti-inflammatory.	[153]
ROS		CC-B-CAPE@CY-NPs hydrogel	PDLSCs, Periodontal tissue	ROS scavenging, Anti-inflammatory, Osteogenesis.	[154]
Thermo-responsive		PF-127@DOX/ALG-MS@PA hydrogel	RAW264.7, MC3T3-E1, Periodontal tissue	Antibacterial, Anti-inflammatory, Osteogenesis.	[155]
PDT	Liquid exfoliation technique	ICG/BPNSs	Periodontal tissue	ROS scavenging, Antioxidant, Antibacterial, Anti-inflammatory.	[156]
ROS	Layer-by-layer assembly	PO/4-BPBA/Mino@COL (PBMC) membrane	Periodontal bone defect tissue	ROS scavenging, Antibacterial, Osteogenesis.	[112]
ROS/Electrical -responsive	Self-assembly *in situ*	BNP-PEDOT-PSF hydrogel	Periodontium in diabetic inflammatory microenvironment	Antioxidant, Anti-inflammatory, Osteogenesis, Angiogenesis.	[157]
Thermo-responsive		PL/LL-37 hydrogel	Periodontium under diabetic conditions	Antibacterial, Osteogenesis, Antioxidant, Immunomodulation.	[158]
PDT/NO		CGP nanoparticle	Apical periodontitis, Alveolar bone defects	Antibacterial, Root canal irrigation, Osteogenesis.	[52]

#### 4.2.1 Antibacterial activity and biofilm elimination

**Fig. 5:**
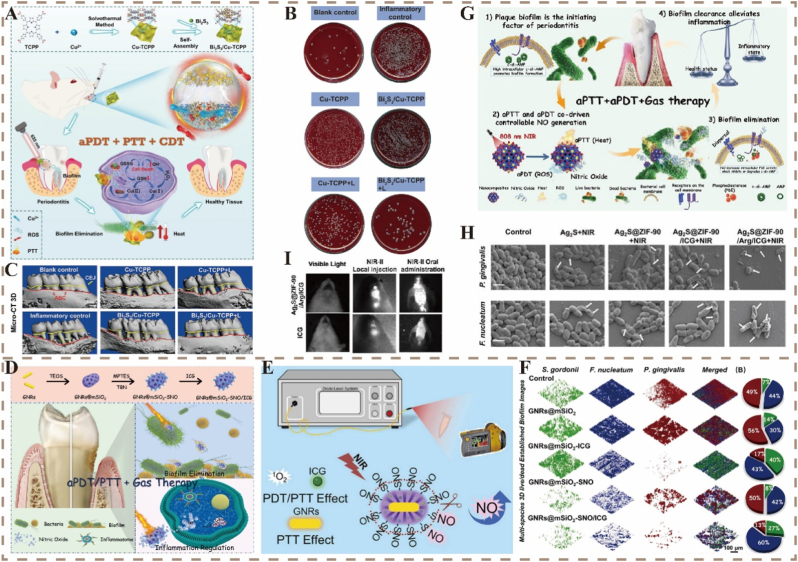
A) Schematic of Bi_2_S_3_/Cu-TCPP nanocomposites in biofilm removal and periodontitis treatment. B) Plate map of bacterial colonies isolated from periodontal pockets. C) Micro-CT 3D reconstructions of maxillary molar regions [159]. Reproduced with permission from Ref. [159] Copyright 2023, Wiley. D) Design and therapeutic function of GNRs@mSiO_2_-SNO/ICG NPs targeting periodontal biofilms and inflammation. E) Schematic of ^1^O_2_ and NO production by GNRs@mSiO_2_-SNO/ICG NPs. F) 3D FISH images of multi-species biofilms [56]. Reproduced with permission from Ref. [56] Copyright 2022, Elsevier. G) Mechanism of Ag_2_S@ZIF-90/Arg/ICG NCs in anti-biofilm, anti-inflammatory therapy via aPTT/aPDT/NO. H) SEM images of *P. gingivalis* and *F. nucleatum* biofilms. I) NIR-II imaging of Ag_2_S@ZIF-90/Arg/ICG NCs and ICG in rats [160]. Reproduced with permission from Ref. [160] Copyright 2023, Ivyspring.

Effective treatment of periodontitis requires a comprehensive approach that integrates the eradication of pathogens, immunomodulation, and functional tissue regeneration. Dental plaque is the primary etiological factor of periodontitis. Therefore, the efficient elimination of multidrug-resistant pathogens and thorough disruption of bacterial biofilms within periodontal pockets are the foundation of successful therapy. In recent years, multifunctional antibacterial nanoplatforms have shown considerable promise in the treatment of periodontitis; they integrate various therapeutic modalities, including aPDT, PTT, CDT, and gas therapy, thereby enhancing antibacterial, anti-inflammatory, and regenerative efficacy. For example, Kong et al. developed a Z-scheme heterojunction composite by anchoring Bi_2_S_3_ nanoparticles onto Cu-TCPP nanosheets (Fig. 5A). This composite significantly increased the NIR light absorption and charge separation efficiency. The combination of PTT and aPDT synergistically increased ROS production under NIR irradiation, effectively disrupting the bacterial biofilm. PTT facilitated the release of Cu^2+^, thereby triggering a CDT effect. The synergistic action of these three modalities significantly improved the bactericidal efficacy against periodontal pathogens. The results of plate count analysis showed that the Bi_2_S_3_/Cu-TCPP nanocomposite effectively eliminated most bacteria under 635 nm laser irradiation (Fig. 5B). Moreover, *in vivo* experiments demonstrated a substantial reduction in inflammation and preservation of alveolar bone structure in animal models (Fig. 5C) [159].

Researchers have developed NIR-triggered nanoplatforms integrating aPDT, PTT, and gas therapy to further enhance periodontal regeneration. A representative example is a core-shell heat-responsive delivery system comprising a noble metal core and a mesoporous silica shell. Qi et al. engineered a multifunctional nanoplatform by co-loading S-nitrosothiols (SNO) and ICG into mesoporous silica-coated gold nanorods (GNRs@mSiO_2_) (Fig. 5D). Under NIR irradiation at 808 nm, the platform generated ROS via PDT to eliminate bacteria and induce hyperthermia to promote the dispersion of biofilms formed by *S*. *gordonii*, *Fusobacterium nucleatum*, and *Porphyromonas gingivalis* (Fig. 5F). The combined effect of heat and ROS triggered the decomposition of SNO, releasing NO gas molecules (Fig. 5E), which in turn inhibited proinflammatory cytokines and the assembly of the NLRP3 inflammasome, resulting in significant antibacterial and immunomodulatory outcomes in deep periodontal lesions [56]. Similarly, a core-shell nanocomposite (Ag_2_S@ZIF-90/Arg/ICG) exhibited triple-modal functionality under NIR irradiation at 808 nm, combining aPDT, PTT, and NO gas therapy (Fig. 5G). This platform achieved potent antibacterial effects along with excellent NIR-II imaging capabilities. After NIR irradiation at 808 nm, the treatment group presented the smallest biofilm area, along with disruption of bacterial membranes and collapse of cellular structures (Fig. 5H). Local injection and oral administration resulted in strong NIR-II signals localized to the injection site or the oral cavity of mice (Fig. 5I) [160]. The use of L-arginine as a NO donor decreased concerns regarding biotoxic byproducts and short half-life, further improving the therapeutic biosafety profile [122,160].

#### 4.2.2 Antioxidation and immune regulation

Eliminating excessive ROS and reprogramming macrophage polarization to restore periodontal immune homeostasis are necessary for suppressing chronic inflammation and promoting tissue repair. Intelligent hydrogel systems with ROS-responsive and immunomodulatory properties can effectively treat periodontitis. Luo et al. developed a dynamic MOF-based hydrogel by embedding magnesium-containing and gallic acid-containing MOFs into a dual-crosslinked network composed of carboxymethyl chitosan, dextran, and 4-formylphenylboronic acid. The imine bonds and boronate ester linkages in the hydrogel responded to pH and ROS, respectively. This system has not only antibacterial and antioxidant effects but also promotes alveolar bone regeneration by guiding macrophage polarization from the proinflammatory M1 phenotype to the anti-inflammatory M2 phenotype [161]. Zhu et al. designed an injectable ROS-responsive hydrogel (HP-PVA@MH/Fe-Que) based on borate bonds formed between hyaluronic acid modified with 3-aminophenylboronic acid and poly(vinyl alcohol) (PVA). The hydrogel encapsulated the antibacterial agent minocycline hydrochloride (MH) and anti-inflammatory Fe (iron)-quercetin nanoparticles (Fe-Que NPs). Under ROS-rich periodontal conditions, the borate bonds were cleaved, accelerating the release of MH and Fe-Que. MH rapidly eradicated bacteria, while Fe-Que NPs activated the Nrf2/NF-*κ*B signaling pathway to scavenge ROS, induce the polarization of macrophages toward the M2 phenotype, mitigate inflammation, and protect hPDLSCs from oxidative stress, thereby increasing their osteogenic differentiation under oxidative conditions [118].

#### 4.2.3 Angiogenesis and periodontal bone regeneration

Successful regeneration of periodontal tissue relies not only on the elimination of biofilms, reduction in inflammation, and the scavenging of excessive ROS but also on the reconstruction of the microvascular network and the simultaneous formation of bone tissue [162]. Through angiogenesis, essential nutrients and growth factors are delivered to newly formed tissue, whereas osteogenesis restores the function of alveolar bone. The coupling between angiogenesis and osteogenesis [163,164] is a central driver of periodontal regeneration and directly influences tissue integration and functional recovery. However, most therapeutic strategies focus on increasing bone volume and often neglect the restoration of vascular function.

To address this limitation, Li et al. developed a multifunctional yolk-shell-structured Cu_2_O@RuO_2_ nanozyme (CRNC). The RuO_2_ shell exhibits ROS-scavenging activity and induces the polarization of macrophages toward the M2 phenotype, effectively mitigating inflammatory responses. The Cu_2_O core simultaneously releases Cu^2+^ in response to pH and ROS stimuli, activating the TGF-*β*/PI3K and HIF-1*α* signaling pathways to promote angiogenesis in human umbilical vein endothelial cells (HUVECs) and osteogenic differentiation of PDLSCs, respectively. *In vivo* experiments confirmed that CRNC not only significantly reduced inflammation but also enhanced alveolar bone regeneration. These findings highlighted that CRNC can effectively coordinate immune modulation, angiogenesis, and osteogenesis, providing a novel strategy for the functional reconstruction of soft and hard periodontal tissues [40].

### 4.3 Multi-modal response strategies for diabetic periodontal regeneration

Diabetes mellitus, characterized by chronic hyperglycemia and systemic inflammation, is a metabolic disorder that significantly increases the risk of periodontal tissue destruction [165]. The rate of tooth loss in diabetic patients is about 11.4 % higher than that in non-diabetic individuals [166]. The hyperglycemic environment promotes the accumulation of advanced glycation end products and ROS, which in turn activate inflammatory signaling pathways, disrupt periodontal homeostasis, inhibit osteogenesis, and promote bone resorption. Additionally, diabetes exacerbates the degradation of periodontal connective tissue by reducing collagen synthesis and increasing collagen breakdown. These pathological changes together contribute to the progressive destruction of periodontal tissues and alveolar bone loss [167]. In such a hyperglycemic and inflammatory microenvironment, the treatment of periodontitis in diabetic patients faces various challenges. Therefore, comprehensive therapeutic strategies that simultaneously address antibacterial activity, glycemic control, anti-inflammatory effects, and tissue regeneration need to be developed to achieve functional reconstruction of periodontal tissues in individuals with diabetes.

**Fig. 6:**
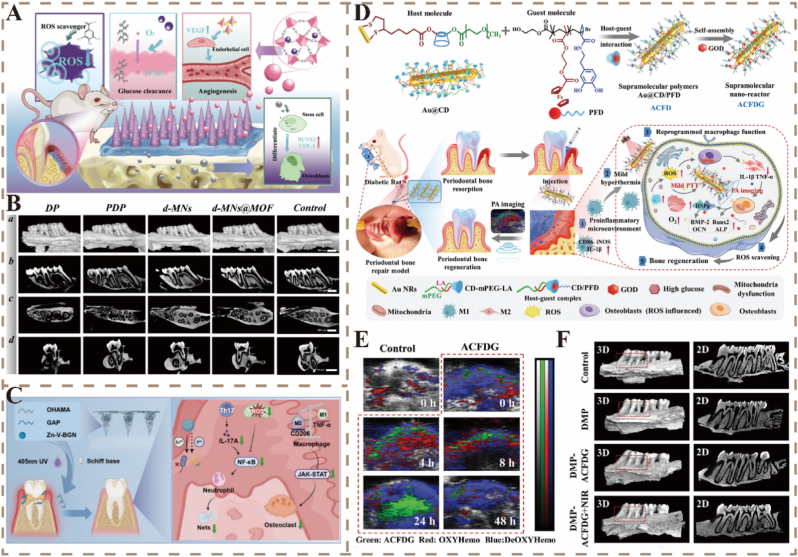
A) Schematic of d-MNs promoting periodontal regeneration. B) 3D reconstruction of the palatal side of the left maxillary first molar [168]. Reproduced with permission from Ref. [168] Copyright 2025, Wiley. C) Illustration of Zn-V-Si-Ca hydrogel microneedles facilitating periodontal repair [169]. Reproduced with permission from Ref. [169] Copyright 2025, American Chemical Society. D) Self-adaptive ACFDG nanoreactor enhances alveolar bone regeneration in diabetic models. E) PA imaging of periodontal tissue after local ACFDG injection. F) Micro-CT images of palatal alveolar bone under different treatments [167]. Reproduced with permission from Ref. [167] Copyright 2024, Wiley.

Eliminating excessive ROS is an effective strategy for controlling inflammation associated with diabetes. Owing to their high drug-loading capacity, controlled-release profiles, and ease of application, hydrogel-based microneedles offer a novel approach to treating periodontitis. These microneedles allow efficient drug delivery directly to gingival tissues, facilitating self-administration by patients. Qu et al. developed a multifunctional double-layer microneedle system (d-MNs), in which the base layer is composed of GelMA loaded with nHA, while the tip is made of hyaluronic acid integrated with gallic acid-functionalized magnesium-based MOFs and loaded with GOx (Fig. 6A). Each component in this system synergistically contributes to the therapeutic effect: gallic acid scavenges ROS and alleviates inflammation, Mg^2+^ is released to promote angiogenesis, GOx catalyzes local glucose under hyperglycemic conditions to reduce glucose levels, and nHA, which has excellent biocompatibility and osteoinductive properties, accelerates osseointegration and bone regeneration. The design ensures that the base tightly adheres to the alveolar bone surface, facilitating the osteogenic differentiation of bone marrow-derived mesenchymal stem cells (BMSCs), whereas the soluble needle tips penetrate the gingival soft tissue to simultaneously achieve anti-inflammatory, hypoglycemic, pro-angiogenic, and osteogenic effects. In diabetic periodontitis models, this microneedle platform significantly increased the regeneration of soft and hard tissues (Fig. 6B), demonstrating strong potential for clinical translation [168]. Another study reported a hydrogel microneedle system based on Zn-V-Si-Ca bioactive glass nanoparticles (Fig. 6C). This system uses the antibacterial and antioxidative effects of Zn^2+^, the insulin-mimicking hypoglycemic properties of V^4+^, the osteogenic activity of V^5+^, and the dual antibacterial and antioxidative functions of gallic acid to improve the diabetic periodontal microenvironment and significantly promote alveolar bone regeneration [169].

Researchers have developed an adaptive photothermal cascade nanoreactor (ACFDG) using a one-step supramolecular self-assembly strategy to address the complex microenvironment of diabetic periodontitis, including hyperglycemia, inflammation, and hypoxia (Fig. 6D). This system comprises gold nanorods (AuNRs), modified *β*-cyclodextrin (*β*-CD), and functionalized ferrocene (PFD), with GOx electrostatically embedded to achieve a high photothermal conversion efficiency of 32.1 %. Under hyperglycemic conditions, GOx catalyzes the oxidation of glucose into gluconic acid and H_2_O_2_. In the presence of PFD, H_2_O_2_ undergoes a Fenton-like reaction to generate **•**OH, effectively mitigating glucose toxicity and initiating a redox cascade. Following NIR irradiation, the photothermal effect of the AuNRs increases enzymatic activity, accelerates ROS elimination, and improves local hypoxia. The system also facilitates macrophage polarization from the M1 phenotype to the M2 phenotype, remodels the immune microenvironment, upregulates the expression of heat shock proteins, and promotes the osteogenic differentiation of BMSCs, collectively contributing to the suppression of diabetes-associated alveolar bone loss. The strong NIR absorption of the AuNRs confers photoacoustic imaging capability, allowing real-time monitoring of oxygen saturation in tissues and providing non-invasive feedback for precise therapeutic control (Fig. 6E). In a diabetic rat model, ACFDG significantly alleviated inflammation and enhanced bone regeneration (Fig. 6F), demonstrating that it can effectively treat diabetic periodontitis. The study presented a novel and precise strategy for the personalized treatment of diabetic periodontitis by integrating photothermal conversion, redox regulation, immunomodulation, and real-time imaging, offering broad prospects for clinical translation [167].

Periodontal tissue regeneration relies on the close collaboration of key processes, including pathogen clearance, modulation of inflammation, glycemic control, angiogenesis, and bone formation. By precisely sensing the dynamic changes within the periodontal pocket microenvironment, smart-responsive nanomaterials enable the on-demand release of drugs and functional factors. This approach facilitates the reprogramming of macrophages toward a pro-repair phenotype, thereby suppressing chronic inflammation. These materials together drive angiogenesis and osteogenesis, restoring the structure and function of soft and hard tissues. Real-time imaging or monitoring technologies provide feedback on tissue oxygenation and inflammation status, offering a novel solution for the precise regeneration of periodontal soft and hard tissues.

## 5 Conclusion and prospects

Smart-responsive nanomaterials, which sense biochemical and physical signals in the oral microenvironment, offer precise molecular interventions for tissue regeneration. The main advantage of these materials is their enhanced targeting capability, which enables selective activation at sites of inflammation or infection, thereby minimizing interference with healthy tissues. These materials also possess environmentally responsive release properties, enabling on-demand drug release in response to microenvironmental changes, which significantly enhances treatment efficacy and safety. Their dynamic adaptability allows them to maintain optimal performance under fluctuating physiological conditions, thereby reducing systemic toxicity. Smart materials can perform multiple functions, such as antibacterial, anti-inflammatory, differentiation-promoting, and angiogenic activities, whereas biomimetic designs enable better adaptation to the complex oral environment. Despite significant progress, several key challenges remain in this field.

First, the oral microenvironment is highly complex and influenced by multiple factors, such as saliva flow, pH fluctuations, microbial competition, temperature variations, and enzymatic activity. However, most smart-responsive nanomaterials are designed under static laboratory conditions, limiting their adaptability to the dynamic conditions present in real-world oral environments. Additionally, the precise delivery, deep penetration, and prolonged retention of nanomaterials in challenging anatomical sites such as periodontal pockets and root canal systems are significant technical limitations, particularly in achieving site-specific targeting and sustained localization. To address these issues, researchers have developed various bioinspired materials with enhanced wet adhesion and structural stability, such as hydrogels based on adhesive proteins or dopamine and nanocomposites derived from natural polymers, which improve the performance of materials in moist and dynamic conditions [170]. Drug delivery systems, including hydrogels, microneedles, and polymeric nanoparticles, can protect therapeutic agents, maintain effective drug concentrations, and reduce side effects [171]. Microneedle patches have shown promising results in animal studies, achieving deep drug delivery and sustained release in gingival tissues while exhibiting excellent tissue penetration and biocompatibility [172]. Future studies should focus on balancing the bioadhesive properties and mechanical robustness of smart nanomaterials while integrating multidimensional responsiveness to oral environmental cues. Such advancements may allow accurate recognition of complex lesions and on-demand drug release, thereby facilitating the clinical translation of smart-responsive nanomaterials in oral regenerative medicine.

Second, current tissue regeneration strategies often rely on the production or elimination of ROS, which possess beneficial and detrimental properties. At moderate levels, ROS can disrupt biofilms, activate immune responses, and increase antibacterial efficacy [173]; however, excessive ROS can induce oxidative damage, impair the functions of tissues, and suppress the activity of stem cells, thereby delaying the repair process. The protumor and antitumor effects of ROS are a paradox, and studies often contradict each other because of differences in the models and methods implemented. For example, ROS can suppress pancreatic tumor initiation at early stages but promote metastasis at later stages [174].

Achieving a dynamic balance between generating and scavenging ROS has become a challenge in this field. In recent years, researchers have focused on smart nanozyme platforms for their ability to mimic natural enzyme activity and modulate ROS homeostasis through spatiotemporally controlled cascade reactions [175]. For example, in acidic wound microenvironments, nanozymes can generate bactericidal ROS via POD-like activity. Under NIR irradiation, these systems can exhibit SOD, CAT, and hydroxyl radical scavenging activities, effectively converting excess ROS (e.g., H_2_O_2_) into O_2_ and alleviating oxidative stress. This strategy to regulate ROS homeostasis can significantly accelerate wound healing by improving local inflammatory conditions and promoting re-epithelialization [176]. Moreover, emerging dynamic ROS modulators offer enhanced targeting capabilities by selectively eliminating cytotoxic ROS while preserving the ROS signals essential for normal cellular function. This selective modulation allows effective treatment of chronic inflammation without disrupting physiological signaling [177] and facilitates targeted production of ROS in tumor tissues while reducing ROS in healthy tissues [14]. Compared to traditional therapeutic techniques, various nanodelivery platforms and nanorobots exhibit superior properties, such as size-dependent effects, surface functionalization, high surface-area-to-volume ratios, and improved targeting efficiency, which collectively enable precise ROS regulation and multi-stimuli-responsive therapeutic approaches [24]. Future strategies may involve developing smart nanomaterial systems with feedback-regulating capabilities, which can continuously monitor ROS levels and automatically adjust them to maintain a safe threshold. Although most studies are in the preclinical stage, promising candidates such as biodegradable nanozymes, natural polymer-based nanoparticles, and free radical scavenging nanocarriers demonstrate significant translational potential and require further investigation and clinical validation.

Third, certain nanomaterials may induce the accumulation of ions or immune rejection reactions during *in vivo* application, thereby compromising their biosafety and clinical feasibility. Although several preclinical studies have assessed the short-term effects of nanomaterials on cellular viability, the hematological system, and organ accumulation *in vitro* or in animal models, our understanding of their long-term toxicity, metabolic pathways, and overall safety in the human body is limited. *In vivo*, metallic nanomaterials may release ions that trigger adverse tissue responses. To mitigate these risks, researchers use materials such as silver and gold, which have higher biocompatibility and lower cytotoxicity [3]. Silver nanoparticles, for example, have inherent broad-spectrum antimicrobial properties. These properties help disrupt bacterial cell walls, inhibit DNA replication, and impair respiratory enzymes [178,179]. Owing to their chemical stability and biological inertness, gold nanoparticles exhibit excellent tissue compatibility and minimal toxicity [180,181]. To address problems regarding immunogenicity, surface modification strategies have been widely adopted. Functionalizing nanomaterials with proteins, lipids, or patient-derived biomolecules [182] can significantly decrease immune activation, prolong systemic circulation, and enhance the efficiency of targeted delivery. The intelligent nano mouthwash with antibacterial and remineralization functions, developed based on the “Interrupting Dental Caries” theory mentioned earlier, has a significantly better caries prevention effect than traditional CHX mouthwash, which verifies its stability and therapeutic potential in the oral environment and shows potential for clinical translation [100]. Achieving a balance between therapeutic efficacy and metabolic safety is a key challenge in the clinical translation of nanotechnology for dental applications. Future studies should focus on optimizing the material composition, engineering responsive surface functionalities, developing controlled degradation mechanisms, and coordinating interactions with the host immune system to increase their potential in oral tissue engineering.

Fourth, large-scale production of smart-responsive nanomaterials needs to be standardized, and challenges such as batch-to-batch consistency and reproducibility need to be addressed. Additionally, the quality control system remains underdeveloped and lacks unified evaluation criteria and testing methods, making it difficult to comprehensively assess safety and efficacy. From a regulatory perspective, current approval frameworks are largely based on conventional drug and medical device regulations, which do not adequately account for the unique characteristics of nanomaterials, such as size-dependent effects and surface functionalization. This regulatory misalignment makes the approval processes long and complex, further limiting their clinical adoption. To address these challenges, researchers need to promote the integration of materials science with oral medicine and foster interdisciplinary collaboration. Future development should be oriented toward practical clinical needs, focusing on oral nanomedicine formulations with functional applicability and scalability, such as oral delivery systems, hydrogels, mouthwashes, and toothpaste formulations, to increase feasibility and value in daily oral healthcare [183]. Periodontal vaccines represent a novel approach to the prevention and control of periodontitis, highlighting the expanding scope of therapeutic strategies [184]. The integration of microfluidic systems with 3D bioprinting has attracted considerable attention as an innovative strategy for the large-scale fabrication of standardized and uniformly sized biomaterials [185]. Therefore, a robust production standard, quality control system, and regulatory approval mechanism need to be established to accelerate the clinical translation of nanomaterials in oral regenerative medicine.

To summarize, smart-responsive nanomaterials can revolutionize dental healthcare by offering precise, personalized, and minimally invasive treatment options. These materials can dynamically regulate the release of drugs in response to changes in the oral microenvironment, enabling multifaceted functions such as antibacterial and anti-inflammatory effects, tissue repair, and bone regeneration. These functions support the development of innovative therapeutic strategies for managing complex oral diseases. As advancements in technology continue and related challenges are progressively addressed, smart-responsive nanomaterials may significantly increase treatment efficacy, decrease side effects, and accelerate recovery, thereby introducing more efficient and precise dental care.

## CRediT authorship contribution statement

**Chenying Cui:** Writing – review & editing, Writing – original draft, Conceptualization. **Jingyu Yan:** Writing – review & editing, Investigation, Data curation. **Lihong Zhou:** Validation, Supervision, Conceptualization. **Yurong Xu:** Validation, Supervision. **Guning Wang:** Supervision, Data curation. **Xiuping Wu:** Investigation, Funding acquisition, Conceptualization. **Bing Li:** Investigation, Funding acquisition, Conceptualization.

## Funding

This work was supported by the 10.13039/501100001809National Natural Science Foundation of China (82470959), Scientific Activities of Selected Returned Overseas Professionals in Shanxi Province (20240016), Shanxi Province Basic Research Program (Free Exploration) Youth Project (202303021212133), Scientific research project of Shanxi Traditional Chinese Medicine Administration(2024ZYYB050).

## Declaration of competing interest

The authors declare that they have no known competing financial interests or personal relationships that could have appeared to influence the work reported in this paper.

## Data Availability

Data will be made available on request.
